# Surgery of the Intestines

**Published:** 1905-03-11

**Authors:** 


### SURGERY OF THE INTESTINES.

**Intestinal Obstruction.**—Barker[1] says that the factors in obstruction which we have to treat surgically may be conveniently arranged under three heads :—(1) Mechanical or anatomical factors ; (2) physical factors ; (3) vital factors. Under the head of "anatomical factors" will come the consideration of the part of the intestinal tract which is the seat of the stoppage. The higher up the obstruction, the earlier and the more acute are the symptoms. All the variations in the form of mechanical cause of obstruction have an important bearing on the treatment, and must be constantly borne in mind. They are relatively the least important of all the factors. They have to be removed, and this is a matter of mechanics and comparatively easy. Of far more importance are the physical changes in the bowel. These include the anæmia at the point constricted, the venous engorgement of the distal parts, the consequent œdema, thrombosis, and frequently sphacelus or ulceration of that portion which are inevitable if there is delay in the treatment. But under the heading "vital changes" come the most important factors of all in stoppage of the bowels. Above an obstruction, say low down, we have much retained fæces and hypersecretion from the gut, swarming with micro-organisms. The bacteria fabricate in time large quantities of toxins, for which there is no way of escape. Consequently they are absorbed into the system, producing profound general depression of vitality, with fever, and often lung and kidney complications. In advanced cases, too, we have to deal with infection of the peritoneum even without perforation ; and, moreover, after an obstruction has been relieved, such peritonitis may start from an unopened bowel soddened with toxins and loaded with putrid material, which, in its paralysed condition, it is unable to get rid of by propulsion. Before operation it is important to empty the stomach by means of a tube. He draws the following conclusions from his reasonings :—1. That too much stress has hitherto been laid upon the mere removal of the mechanical stoppage, and not enough upon the evacuation of the putrid material which is killing the patient.. 2. That the older methods of emptying the bowel by establishing an artificial anus are unsatisfactory, as the bowel is in a state of paresis, or complete paralysis, and only expels its contents very slowly, and until it is cleaned out the cause of the paralysis remains in great part. 3. That the best course in many of these cases, probably in all that are still within the reach of any operative procedure, is to resect the tract of paralysed and distended bowel at once. This would not, of course, apply to recent cases where the bowel is not seriously damaged by the retained fæces, and is manifestly sound at and above the seat of obstruction. In most cases he believes in closing the abdomen without drainage unless there is general peritonitis. The patients should sit up in bed from the first so as to overcome any tendency to hypostatic pneumonia. They should begin at once to take a mixture containing 10 grains of carbonate of bismuth, as this is the best intestinal antiseptic. Nutrient enemata and instillation under the skin of normal saline solution or 5 per cent. solution of glucose is recommended as a routine. Half a litre morning and evening may be given if necessary. Interesting cases are recorded by Morison,[2] Wallace,[3] and Fostner.[4]

**Exclusion of the Intestine** means, says Moynihan,[5] the rendering inactive any part of the intestinal canal by surgical means. The following forms of intestinal exclusion are recognised :—1. Entero-anastomosis—the "short-circuiting" of English writers. 2. Entero-anastomosis with constriction by suture of the part distal to the upper opening. 3. Unilateral exclusion, when the gut is divided completely across and the proximal end is implanted into the side of the distal. The distal end may be closed or left open. 4. Bilateral exclusion, when the bowel is divided at two places and the intestinal channel is restored, leaving a segment of the bowel detached. Of this segment one end may be left open and stitched to the skin, or both ends may be closed when a fistula exists in the bowel lying between them. In all operations of this kind it is desirable to open the abdomen if possible in the middle line. The incision should be made at a distance from any fistula or any skin involvement. It is desirable to work from some fixed point, the cæcum by choice or the duodeno-jejunal flexure. When the proximal and distal portions of the bowel have been recognised, the former is divided completely across. The method of dividing it depends upon the purpose of the operator. If an end-to-end or end-to-side anastomosis is to be performed it is to be divided between two clamps, the blades of which are sheathed with rubber tubing. If the ends are to be closed, and a lateral approximation is to be made (which is the method of choice when the small intestine has to be united to the larger), then the bowel is crushed by forceps, which divide all the coats except the peritoneum, two catgut ligatures are applied in the groove left when the forceps are removed, and the bowel divided between the ligatures. The stump on each side is then buried by a continuous suture. The operation of exclusion of the intestine is chiefly necessary in cases of chronic inflammatory disease, probably tuberculous, involving the bowel and leading to external fistula ; in cases of inoperable carcinoma of any part of the large intestine, especially of the cæcum and ascending colon ; and in rebellious cases of muco-membranous colitis. From what has been said it will be recognised that unilateral exclusion, save in cases of disease affecting the lower end of the ileum, offers no advantages over lateral anastomosis, and that bilateral exclusion affords the best means of dealing with certain diseases, chiefly tuberculous and malignant, in the cæcum and colon, and with fistula between the small intestine and the bladder or vagina.

**Intestinal Perforation in Typhoid Fever** has been investigated by Haggard,[6] who deduces the following facts from a statistical study of 295 operative cases: 1. It occurs more often in men than women —80·9 as compared with 19·1 per cent. It is, like hæmorrhage, rare in children. 2. It occurs in about 2·3 per cent. of all cases of typhoid fever. 3. 3·91 per cent. occur in the first week, 20·19 per cent. in the second week; 38·94 in the third week; 14·90 in the fourth week; 9·13 per cent. in the fifth week; 5·75 in the sixth week; 7·21 per cent. from the seventh to the eleventh week, and it has been observed as late as the one hundredth day. 4. It naturally occurs more frequently in severe attacks, but may occur in mild attacks, and it may be the first real symptom of so-called walking typhoid. 5. It occurs in the ileum in 95·5 per cent., usually within 18 inches of the cæcum, always within three feet. In the large intestine in 12.9 per cent.; and is most often situated in the ascending, transverse, and descending colon, sigmoid and rectum in the order named. It may occur also in the appendix, Meckel's diverticulum, and jejunum. 6. The perforation is single in 84 per cent. There may be two or more perforations. Cases with diarrhœa and tympany are more likely to have perforation. Six out of 30 cases occurred with hæmorrhage (Osler). 7. The death-rate given by Murchison is 90 per cent. to 95 per cent. Osler says he cannot state a single case in his experience that had recovered after perforation had occurred. The author prefers the right iliac incision unless there is general peritonitis, and records three successful operations by suture. Drainage by the vagina is preferable in women. Lumbar puncture and drainage by tube and gauze is expedient in men. Thornton and Sanders[7] also record a successful operation case. Le Conte[8] draws attention to the fact that rupture of mesenteric glands in typhoid fever may simulate intestinal perforation.

**Embolism and Thrombosis of the Vessels of the Mesentery.**—Moynihan[9] says that in this condition a diagnosis of intestinal obstruction is generally made, and it is only at the operation or at an autopsy that the exact condition of affairs is revealed. Gerhardt considers the following signs as necessary to a diagnosis:—1. The presence of a source for an embolus. 2. Copious intestinal hæmorrhages, not to be explained by disease of the bowel wall, or by impediment to the portal circulation. 3. A rapid and marked fall of temperature. 4. Colicky pains in the abdomen. 5. Later, distension of the abdomen and the presence of free fluid. 6. The simultaneous or previous occurrence of embolism in other parts. 7. The occasional presence of a tumour in the abdomen due to the infiltration of the mesentery by blood. Not all of these signs are present in any one case, but the existence of some of them is necessary to the making of a diagnosis. The prognosis is very bad, the mortality being 94 per cent. After the abdomen is opened the gangrenous gut is withdrawn from the abdomen, the bowel is divided and the whole involved part is removed. In all cases drainage of the intestines must be ensured—as by placing two Paul's tubes in the divided ends of the bowel.

[1] Lancet, Sept. 17, 1904, p. 809. [2] Edin. Med. Jour., August, 1904, p. 130. [3] Scot. Med. and Surg. Journ., April, 1904, p. 289. [4] Lancet, July 23, 1904, p. 208. [5] Lancet, Oct. 8, 1904, p. 1010. [6] Med. Rec., July 16, 1904, p. 89. [7] Lancet, June 18, 1904, p. 1723. [8] Med. Rec., June 18, 1904, p. 1023. [9] Prac., October, 1904, p. 538.

